# Concepts, Beliefs, and Traditional Treatment for Childhood Seizures in a Quilombola Community in Northeastern Brazil: Analysis by the Discourse of the Collective Speech

**DOI:** 10.3390/ijerph18041875

**Published:** 2021-02-15

**Authors:** Izabel Cristina Santiago Lemos de Beltrão, Yasmin Ventura Andrade Carneiro, Gyllyandeson de Araújo Delmondes, Luiz de Beltrão Lima Junior, Marta Regina Kerntopf

**Affiliations:** 1 Department of Nursing, Universidade Regional do Cariri, 1161 Coronel Antônio Luíz St, Crato, CE 63105-010, Brazil; yasmin.ventura@urca.br (Y.V.A.C.); mrkerntopf@outlook.com (M.R.K.); 2 Natural Products Pharmacology Laboratory, Department of Biological Chemistry, Universidade Regional do Cariri, Crato, CE 63105-010, Brazil; gylyandeson.delmondes@urca.br; 3 Department of Zootechnics, Instituto Federal do Ceará, Campus Crato, CE-292 Gisélia Pinheiro, St. Crato, CE 63115-500, Brazil; beltrao.junior@ifce.edu.br

**Keywords:** beliefs and attitudes, epilepsy, seizures, traditional medicine

## Abstract

Background: Non-pharmacological therapy related to traditional, magical, and/or religious treatments for managing recurrent and non-recurrent seizures in children persists in several traditional communities. The research aims to investigate the concepts, beliefs, and types of traditional treatments used for cases of seizures in children reported by residents of a quilombola community. Methods: The research took place in the quilombo community Sítio Arruda, Ceará, northeastern Brazil. The study population consisted of 19 participants, including healers, prayers, and midwives. Applied a socioeconomic form and a semi-structured interview script. For data analysis, the Discourse of the Collective Speech (DCS) technique was used. Results: For the questions asked, a total of 14 central ideas were found. The most prevalent was seizure is the most common type of disease in children (50.0%); The seizure occurs because of the fever (42.0%); In the community, we treat and prevent seizures with the use of plants (63.2%). Conclusions: The present study’s results addressed relevant issues that include valuing and understanding the traditional knowledge of the community, access to health services, and the need for clarification actions about seizures.

## 1. Introduction

Seizures are the most common neurological disorder during childhood. Approximately 10% of the world population has the possibility of having a type of seizure disorder, of which 50% will occur during childhood and adolescence, with a higher risk in the age group of neonates (0–29 days) and infants (1 month–11 months) and 29 days) [1]. 

Symptoms related to seizures are potentially frightening and inherently dramatic for family and friends. In the long term, they can represent epilepsy—severe neurological damage—, associated psychiatric disorders and relevant impact on the quality of life, causing withdrawal and social isolation, strengthening the stigma that permeates seizures [2]. 

Currently, the treatment objective is, in the case of acute seizures, to treat the trigger-ing cause of the seizure, such as fever and infections, reducing the risk of neurological damage. In contrast, chronic seizures treatment aims to provide a good quality of life using antiepileptic drugs [3]. 

In this context, non-pharmacological therapy related to traditional, magical, and/or religious treatments also stands out. For example, many studies showed flora resources as the non-pharmacological treatment of convulsive disorders in traditional communities [4,5]. Also, the association of convulsive crises with hidden or bad influences permeates several populations’ imagination, especially when considering traditional or culturally different communities [6]. 

Some of these practices currently persist in several traditional communities, being motivated by multiple factors related to the high costs of biomedical treatment (transportation, consultations, and medications), lack of confidence in the effectiveness of long-term pharmacological therapy, presence of adverse effects related to antiepileptic medications and a strong influence of beliefs and caregivers in these rural communities [7,8]. 

Beliefs that associate seizures as a form of divine punishment, as a type of curse or demonic possession, as an infectious disease, or as a psychiatric disorder are recurrent in different parts of the world and direct ineffective therapies, cause stigma, abandonment, and child neglect, isolation social and important socioeconomic impacts [9,10,11,12]. In this sense, it is highlighted that a misconception anchors many of the beliefs cited about the etiology of seizures [13]. 

Given the above, the remarkable scientific value in the development of research that seeks to rescue the traditional knowledge established related to the management of pathological conditions is emphasized, assessing the degree of influence they determine in health care [14]. 

Therefore, considering the possible impacts related to the risk of neurological damage and the quality of life of children who have suffered or suffer from seizures, the stigma that accompanies this disorder, the magical thinking associated with it by many communities, and the difficulties in accessing treatment conventional treatment for rural populations, the present study aimed to investigate which concepts, beliefs, possible stigmas and types of traditional treatments used for the management of acute and/or chronic childhood seizures in a quilombola community located in the area of the state of Ceará, in the northeast of Brazil.

## 2. Materials and Methods

### 2.1. Study Location and Participants

We chose the community of Sítio Arruda, located in the municipality of Araripe—Ceará—because it is a community remnant by quilombo certified by Brazil’s Ministry of Culture [15]. Besides, while conducting ethnobotanical studies at Sítio Arruda [16], the presence of relatively frequent neurological disorders was mentioned among some community members, most notably seizures in children, raising interest about how Sítio Arruda understood and treated within the scope of practices cultural health cases of childhood seizures. 

The remaining quilombo communities derived from the old quilombos that housed fugitive slaves who resisted slavery, established in approximately 1535, lasting until 1888 in Brazil. In this way, descendants of African slaves form the remaining communities. Quilombola populations and indigenous populations, among others, are traditional populations in Brazil, as they have their forms of social organization, occupy and use territories and natural resources as a condition for their cultural, social, religious, ancestral, and economical reproduction [17]. 

In Brazil, there are thousands of self-proclaimed remnant quilombo communities, however, only about 154 have been titled and certified after anthropological studies and technical reports. The entire certification process, which guarantees the legal right to ancestral land use and other prerogatives, can take many years. 

In 2017, community leaders Sítio Arruda reported that 35 families lived in the community, with one or more families living in the same house. The number of residents in the community is approximately 150 members. There is no basic sanitation in the community, and many causes of infectious diseases, systemic arterial hypertension, chronic musculoskeletal pain (possibly due to work activity), and mental disorders, in addition to reports of neurological disorders.

Considering that the study’s aim was directed at a specific population, with a method that includes a qualitative approach focused on a particular reality, the sample chosen was non-probabilistic and contacted the research subjects directly in the community. The study population consisted of people of both sexes, living in the quilombo community of Sítio Arruda, aged between 18 and 85 years old, mothers, other family members and/or guardians of children who had seizures for at least a single time, as well as residents of the community with differentiated traditional knowledge. Examples of community residents with differentiated traditional knowledge are Such as healers, prayers, herbalists, and midwives, totaling a sample of 19 participants. 

As exclusion criteria, we defined people suffering from alopsychic and autopsychic disorientation, psychiatric disorders that hinder their understanding of reality, such as neurosis, schizophrenia, and manic-depressive disorders. Also, users under the effect of psychoactive substances that cause changes to a greater or lesser extent in motor and/or mental functions, as reported by the family and/or other community members. 

The research sample consisted of 19 participants, 17 (89.5%) female informants, and two (10.5%) male informants. There was no refusal or withdrawal of residents contacted in the community invited to participate in the study. Most of the research participants were 46 to 59 years (47.4%), and the most reported marital status was married (52.6%). Considering the level of education, nine (47.4%) respondents reported not having completed elementary school. There was still an expressive number of the sample declaring themselves out of school, a reality reported by eight (42%) participants. 

These data show a panorama of low schooling in the community, regardless of the age group considered. In this sense, the prevalent profession is farming, representing 57.9% of the sample. Regarding residence time in the area, 13 participants (68.4%) reported living there for a period greater than or equal to 30 years. 

### 2.2. Instruments and Procedures for Data Collection 

At first, we applied the “rapport” technique through initial contact with the community leader. Subsequently, in the second moment, after authorization and indication from the community leader and a favorable opinion from the competent legal bodies—respecting the ethical and legal precepts of research involving human beings—the first study participants were contacted at their homes. Indicated others with the potential to contribute to the study, a technique characterized as “snowball” [18]. First we applied a form for characterizing interviewees’ socioeconomic status. Then, we conducted a semi-structured interview with three questions intended to investigate the concepts, magical-religious practices, and traditional treatments related to sporadic or preventive management of seizures, whether acute or chronic. We conduct the data collection between October 2017 and January 2018. Each interview lasted, on average, 45 min. A researcher with experience in ethnographic studies and nursing training conducted the interviews in Portuguese. A research assistant registered the photographic records and recordings of interviews. However, the community’s initial contact and subsequent visits for notes in the field diaries occurred since March 2016.

### 2.3. Data Analysis 

For data analysis, using the Discourse of the Collective Speech (DCS) technique. In research with the DCS, the thought is collected by individual interviews, from open questions, to rescue the essence of plural opinions, which result in a set of collective discourses, or DCS. The DCS proves to effectively express a community’s thinking on a given topic [19]. Through this proposal to tabulate qualitative data from a verbal nature, it becomes possible that each individual interviewed in the study can contribute to the construction of collective thinking [20]. According to Lefèvre and Lefèvre [19], the DCS is based on the hypothesis that individuals in society share beliefs, values, and social representations.

The method also allows quantifying frequencies of recurring thoughts, being considered a mixed method. Developed a methodological process capable of managing an organization of verbal expressions from social research that use open questionnaires for data collection, applied to specific groups, with converging characteristics for one or more factors considered for the research. 

This methodological process involves the operators: Central Idea, Anchorages, Key Expressions, and DCS. The key expressions (KE) are literal transcriptions of the participants’ expressions that reveal the essence of the announced thought, synthesizing a Central Idea (CI) that guides the entire speech. The anchorages (AC) are generic and explicit statements to endorse an opinion. An informant will not necessarily express a CA in his speech.

Therefore, we formed the DCS from the combining the KE of different participants’ discourse that indicates a certain CI or AC. The formula below illustrates the DCS formation process [21]:

Schematic representation A: DCS 1 = [KE (n2) + KE (n5) + KE (n8) + KE (nx)]^CI^

Schematic representation B: DCS 2 = [KE (n3) + KE (n7) + KE (n9) + KE (nx)]^AC^

where DCS = Discourse of the Collective Speech; KE = Key expression; CI = Central Idea; AC = Anchorage; n = study participants who presented the same CI or AC in their speech.

### 2.4. Ethical and Legal Aspects 

The researchers submitted the project to Plataforma Brasil (PB), which is therefore forwarded to the Research Ethics Committee of the Universidade Regional do Cariri (URCA), obtaining approval record number: 1367311. The study was registered in the Sistema Nacional de Gestão do Patrimônio Genético e do Conhecimento Tradicional Associado (SISGen), with code: A52C550.

## 3. Results

### 3.1. Discourse of the Collective Speech

#### 3.1.1. Seizure Concepts

When asked about seizures, the symptoms most frequently present in the imaginary and/or observed directly by the participants were clonic movements; unusual orbital motion; loss of postural control; salivation; an unusual movement of the head; disorientation and mental confusion; loss of consciousness; bladder incontinence; loss of muscle tone and amnesia. The most reliable words to refer to seizures in the community was: attack, child shaking, and head disease, with the word attack expresing the greatest number of characteristics as described by the interviewed residents of Sítio Arruda.

The next tables, the data referring to the Discourse of the Collective Speech (DCS), elaborated through the interviewees’ oral expressions, from the four questions formulated for the study, follow. The table shows the relationship between the Central Idea (CI), the proportion of responses according to the participants’ opinion, and the DCS for each CI identified. Each question was to the participants, according to the order adopted for the interview in the community. Table 1 below shows the DCS for question 1: In your opinion, what is a seizure?

For question 1, four CIs were found, no anchorages were identified (AC), and the prevalent CI was “*Seizure is a type of disease more common in children*” mentioned by 50% of respondents who answered the question.

#### 3.1.2. Causes of Seizure in Children

Regarding the causes of seizures in children, the results are shown in Table 2.

For question 2, six CIs were found, no anchorages were identified (AC), and the prevalent CI was “The seizure occurs because of fever” mentioned by 42% of respondents who answered the question.

#### 3.1.3. Treatments for Seizure in Children and Traditional Medicine

Concerning some treatments performed in the community for the management or prevention of seizures in children, the results are shown in Table 3.

As shown in Table 3 there were four CIs for question 3. The most recurrent CI was “*In the community, we treat and prevent seizures with the use of plants*,” expressed by 63.2% of the sample. Also, question 3 did not identify AC.

## 4. Discussion

### 4.1. Health Care Practices at the Quilombo Community 

In 2017, in the quilombo community Sítio Arruda—CE, there were approximately 150 members. The average number of children of the survey participants was approximately four per family, according to the survey conducted with the residents of the community interviewed for the study. Of the 19 participants, two female informants reported not having children. Another relevant factor concerns access to health services. There are no basic health units within the community or appropriate support points for residents’ extensive health care actions, making access limited. Few residents reported seeking primary care or hospital services, including managing chronic health conditions or even sporadic or recurrent seizure episodes, as mentioned in the interviews. 

Concerning the cases of epilepsy in the Sítio Arruda community, at least three children were undergoing continuous drug treatment, as reported by family members, and confirmed from medical prescriptions presented by those responsible for the researchers, for at least one child, with suspected psychiatric comorbidity. There was no imaging, laboratory tests, or medical opinion to certify the diagnosis [22]. It is believed that this fact corroborates the difficulty in establishing accurate and reliable diagnoses of epilepsy cases in rural communities, which can be at odds when compared to the reality of urban or semi-urban areas [23]. It is also noteworthy that non-recurring cases of seizures in children in the community were reported more frequently. Some seizures affected more than one child in the same family, including a history of seizures in the parents and/or other family members of the children. 

These findings are consistent with current research, pointing to specific genes triggering recurrent seizures or epileptic diseases in childhood [24]. The data related to the socioeconomic profile presented was characterized by the predominance of the female sex and the “married” marital status, with a concentration of the age groups of elderly and mature adults. Also, the prevalent profession was that of a farmer, and the sample population was characterized by low schooling and length of residence in the area over 30 years [16,25]. The sample included one healer, two mourners, and two midwives, identified by residents and according to self-declaration. 

### 4.2. Concepts and Definitions of the Word Seizure 

When asked about the meaning of the word seizure, 50% of the participants expressed an opinion associated with childhood seizures. Some reported witnessing seizures of their children, nephews, and/or grandchildren. The most common symptoms in the participants’ imagination were clonic movements, unusual orbital movement, and loss of postural control.

About the symptoms most mentioned in the speeches, in the study by Deresse and Shaweno [26], people identified the manifestation of the epileptic episode as linked to the following symptoms: convulsion (probably clonic movements); loss of consciousness; foaming at the mouth; roll your eyes up; transient changes in behavior and periods of amnesia. A similar result was found in Kabir et al. [27] research, with the symptom’s inclusion: “biting the tongue.” For the findings of Ezeala-Adikaibe et al. [28], the term “Jerking of the body” was specified, referred to by more.

In fact, seizures are common in children. According to Lefèvre [29], some reasons have been reported in the medical literature for a long time that justifies this fact, such as deficient protection of infantile nerve cells; calcium deprivation to the nerve cell at the expense of developing bone tissue, and lack of synchronism between the functioning of the nervous system and the functioning of the endocrine glands. 

### 4.3. Causes of Seizure in Children 

Regarding the causes of the seizure, the recurrent idea in the ‘respondents’ imagination is fever, since 42% of the sample reported that seizures occur due to febrile episodes [30]. This association is not surprising, considering that febrile seizure is classified as the most prevalent seizure type in the pediatric population. However, these seizures generally do not represent long-term neurological complications associated with epileptic diseases [31]. 

At least 21.1% of the sample at Sítio Arruda was unable to explain the causes of seizures [32]. Simultaneously, the same percentage reported numerous factors related to seizures: sunbathing, adverse effects of medications, intestinal infection, childbirth complications, and mental disorders [33,34]. Genetic factors have also emerged in some speeches (10.5%), although these factors are poorly understood. (10.5%) [35]. Regarding the causes of spirituality and religiosity, 15.8% of the interviewees identified the “lack of faith” and “brokenness” as a type of spell as the cause of the convulsion [36]. 

About supernatural causes, “quebranto” as mentioned by the residents of Sítio Arruda, would be more related as “evil eye”, the most culturally accepted cause for the beginning of epilepsy in regions of Saudi Arabia, and commonly referred to in Asia, the Middle East and part of Europe as associated with seizures [35]. 

### 4.4. Treatments for Seizures in Children: Isolated and Recurrent Episodes 

Regarding the treatments used in the Sítio Arruda community for seizure cases, either isolated and/or recurring episodes, to prevent or treat the child after the crisis, treatments associated with religion, spirituality, and plants. In the first three central ideas that emerged from the speeches, it was possible to point out the treatment involved: prayer and blessing (15.8%), promises (10.5%), and sympathies (21.1%). It is important to note that there is no clear distinction between healers and prayers in the community regarding prayers and blessings. This is clear in the speech: “*there are the healers here. An aunt of mine even prays. There is a girl there who only returned when she prayed*” (see Table 3). 

This distinction is considered relevant for some studies focused on rustic or traditional medicine [37]. For example, in Araújo [38], every healer can provide prayer, but not every person who prays has the gift of blessing, as perceived in some communities. At Sítio Arruda, it is recognized only by the interviewees that some community members present gifts related to healing through prayer, referring to these members, all female, as healers and/or prayers, the terminology “rezadeiras” being more common. Some interviewed reporters called themselves midwives as well. Following the tradition of their ancestors, prayers do not say out loud prayers, considered secret. 

Another relevant aspect concerns sympathies or rituals, followed by some members to prevent the child from having a convulsive episode, mentioned two specific sympathies, both related to fire: “You take the baptism outfit, white, take a strip […] and it burns, it’s for […] children not to have this disease […]. [But] if the child has the attack, the whole disease […] stays on the clothes, you take off the clothes before the child returns and burn all the clothes” (see Table 3). It is known that forms of treatment are causally related to how the community perceives seizure disorder or isolated seizure episodes. 

This relationship of fire-extinguishing power to treat or cure seizures remains in the records of other African and Nordic peoples’ beliefs and cultural practices [39,40]. These practices aim to “send the disease away,” as was said at Sítio Arruda. Other studies also point to traditional treatments that highlight religious and ritualistic aspects for treating seizures in children [41]. 

Regarding the other form of traditional treatment, the research participants also talked about the use of medicinal plants at Síito Arruda. Community members mentioned the recurrent use of plants (63.2%). In fact, plants’ use was the most recurrent type of imaginary treatment in the study population to treat seizures. In the following speech, we can see this: “The people give more plants here. We have all kinds of plants, to use when you need them […] we also buy them at the fair nearby, many children get cured with plants on the farm. Here, people use even more natural remedies. There is a plant that cures everything […], and there is a plant for that too [convulsion].” (see Table 3). 

In the speeches, some plant species by popular name were listed, totaling twenty species. In this regard, it is reiterated that plants’ use for the treatment of seizures is a recurrent practice in traditional medicine and reported in several countries [42,43,44]. Therefore, according to Kakooza-Mwesige [5], the reasons linked to the practice of using plants include the presence of epilepsy resistance to conventional medication, the side effects associated with anticonvulsants, and the belief in the safety and effectiveness of natural resources. 

The author also reiterates the need to consider using this culturally accepted treatment, a phenomenon observed massively, especially in the reality of developing countries [16,25]. Thus, seizures are characterized as a neurological condition little understood by the general population [13,45]. In fact, several misunderstandings and stigmas still permeate communities’ imagination around the world [46,47], especially in developing countries [14,48]. This expresses high rates of low education and limited access to health services, significantly impacting the effectiveness of management and/or treatment and quality of life of people with seizures or diagnosed with epileptic disease [49].

As limitations related to the present study, we can mention the lack of knowledge of some contacted members regarding the investigated neurological condition and possible treatments. Besides, due to the scarce search for health services and difficulties in accessing these services, it was not possible to establish diagnoses accurate reports of epileptic diseases through reports or medical opinion, using only the residents’ reports, based on the classic symptoms referred to the seizures and medical prescriptions given to children, in the case of children with epilepsy.

## 5. Conclusions

The present study results, conducted in the Sítio Arruda quilombola community, made it possible to outline the concepts, beliefs, and types of traditional treatments used for the management of seizures in childhood. Simultaneously, shedding light on relevant issues that include the appreciation and understanding of traditional knowledge from the community, access to health services—effective and resolving—and the need for clarification actions about convulsive crises. 

In part, these goals can be achieved through dialogue and collaboration of people with differentiated traditional knowledge in the community, by improving facilities and health supplies, actions focusing on community clinic and health education practices with the potential to clarify doubts and dispel stigma.

In this way, during the research, we talked to community leaders about how the university could act in articulating truly effective, broad, and continuous actions for the prevention of preventable health problems and in the rehabilitation of non-preventable health problems, also encompassing mental disorders and neurological disorders present in the community. 

In this context, based on the principles of the National Policy for Integrative Health for Rural and Forest Populations, the University Extension Project Promotion of Health and Sustainability in Quilombola Communities emerged. Since 2019, the aforementioned project has developed health care and education actions in the Sítio Arruda community, in partnership with the Municipal Health Departments, Health Residencies, University, and Federal Education Institutes. 

Scholars and volunteer professionals from the project regularly carry out dozens of consultations, exams, quick tests, and educational activities in the community, seeking to demystify diseases and pharmacological treatments, directing practices focused on promoting and preventing health the rational and safe use of resources natural. 

The present research also awakens public authorities’ responsibility and competent bodies in guaranteeing the constitutional rights of the traditional peoples of Brazil, including access to health services in all spheres of complexity. 

We also believe in the intersection and responsibility of academic institutions in this process, guaranteeing social return through coordinated actions that strengthen and expand the implementation of existing public policies and encourage research to collaborate to formulate strategies in line with traditional communities’ reality also effective.

## Figures and Tables

**Table 1:** Relationship between central idea (CI) of question 1, proportion of responses according to research participants and DCS for question 1.

Question 1: In Your Opinion, What Is a Seizure? ^#^			
Central Idea (IC)		Community Residents	
		*n*	%
A	The Seizure is the most common type of disease in children	9	50.0
B	The Seizure is a type of disease caused by a neurological condition	5	27.8
C	I do not know how to define what is a seizure	5	27.8
D	The Seizure is a type of epilepsy	2	11.1
Total Informants = 18 * ^▲^			
**Discourse of the Collective Speech**			
DCS—Central Idea A: This seizure is like an attack that the child has, a disease, we see it more in a small child, but there is a big boy with it [seizures]. Here in the community, I know that there are children with this problem, only you walk around that you find [in the community]. My niece was trembling all over, struggling on the floor. She lost her senses. I was afraid to see because we think the child is dying. Even today, she has this problem. She needs to take medicine and everything.			
DCS—Central Idea B: I think this seizure is an attack. It is a problem in the head, a disease in the head. I know that. When the person falls to the ground and is struggling, the person can pass out and forget things because it affects the head, right? As is the name [pause], I forgot now, but the person has the seizure, the attack as we call it because he has a problem with his head.			
DCS—Central Idea C: I don’t know. We have a few education here. I can’t tell you what this [pause] seizure is. It’s difficult, I don’t know how to explain it, but I know some children at Sítio Arruda who have this problem [seizure] that you talked about, but talking straight as it is, I really don’t know!			
DCS—Central Idea D: The seizure is the same as [pause] epilepsy, but the seizure is more serious than epilepsy because, in epilepsy, we do the treatment at home, everything is normal. Thus, the strong attack of the seizure is more dangerous. The person may not return from the attack, so he needs to see the doctor later. For epilepsy, it will depend only if the person does not regain consciousness.			

* A participant may present more than one CI in his speech. For this reason, *n* > 18 because the number of times that a given CI was pointed out in the participant’s speech is counted. # Adjusted for the wording of some questions at the time of the interview to allow a better understanding of the community’s residents. ^▲^ One sample participant did not answer question 1.

**Table 2:** Relationship between central idea (CI) of question 2, proportion of responses according to research participants and DCS for question 2.

Question 2: In Your Opinion, Why Do Some Children Have or Have Seizures? ^#^			
Central Idea (CI)		Community Residents	
		*n*	%
A	The seizure occurs due to numerous factors	4	21.1
B	The seizure occurs because of quebranto ^■^	3	15.8
C	The seizure occurs because of fever	8	42.0
D	I cannot explain why the seizure occurs	4	21.1
E	The seizure occurs due to a lack of faith	3	15.8
F	The seizure occurs due to genetics	2	10.5
Total Informants = 19 *			
**Discourse of the Collective Speech**			
DCS—Central Idea A: This disease [seizure] happens for several reasons. Some children get seizures because of the sun. Some children get it because of medicine. Some medicines are nasty for the baby. My daughter once got sick because she ate spoiled food and attacked her, but she never had [seizure] again. It was just once, never again! There is a child who gets it right when he is born! The son of the woman who lives near here was born with this problem. He was born sick. He’s a child who can’t be bored. He’s nervous!			
DCS—Central Idea B: I think there are many bad people, see the child and put the quebranto, the evil eye. But some people put [spells] and do not know. The person does not hurt, so the child is only good with prayers; only this disease comes out with prayers [seizure]. It is good to have an Arruda [plant] in the house because the child is protected from the quebranto. It really protects.			
DCS—Central Idea C: Because of fever, extremely high fever, it happens more in babies, it is dangerous. When the fever rises a lot, you must go to the doctor, because it gives seizure, the child may have a head problem [neurological sequelae] too, he cannot let the fever rise. I’ve seen cases like that. My nephew had it. He got all limp. It was because of the fever; he got the flu and gave him a fever.			
DCS—Central Idea D: I don’t know. I have no idea, the people here [Sítio Arruda] say so much, I can’t tell you why the child has this disease!			
DCS—Central Idea E: I also think it is lack of faith, lack of prayer, then there is this disease [seizure], the child is weak, needs prayers to be well, but you must have faith. Otherwise, it will not do!			
DCS—Central Idea F: I think it already comes from the parents […]. Look, my granddaughter, for example, her grandfather was like that, her father spent a lot of time having these attacks [seizures], and she was born that way.			

* A participant may present more than one CI in his speech. For this reason, *n* > 19 because the number of times that a given CI was pointed out in the participant’s speech is counted. # Adjusted for the wording of some questions at the time of the interview to allow a better understanding of the community’s residents. ^■^ Quebranto: the regional word, typical of the Northeast of Brazil, means a type of spell, evil eye. The quebranto can be put out of envy or extreme admiration.

**Table 3:** Relationship between central idea (CI) of question 3, proportion of responses according to research participants and DCS for question 3.

Question 3: What Are Some Treatments Performed in the Community to Manage or Prevent Seizures in Children? ^#^			
Central Idea (CI)		Community Residents	
		*n*	%
A	In the community, we treat and prevent seizures with prayer and blessing	3	15.8
B	In the community, we treat and prevent seizures with promises	2	10.5
C	In the community, we treat and prevent seizures with sympathy	4	21.1
D	In the community, we treat and prevent seizures with the use of plants	12	63.2
Total Informants = 19 *			
**DISCOURSE OF THE COLLECTIVE SPEECH**			
DCS—Central Idea A: Thus, there are the healers at Sítio Arruda. An aunt of mine even prays. There is a girl there who only returned from the attack when she prayed. The girl never went to the hospital, and she was praying! Some children are only well when they pray. Some prayers are for the child to have no more [seizure].			
DCS—Central Idea B: Promise! I make a promise to every saint.			
DCS—Central Idea C: There are some sympathies that the people here do. There is that of baptism. You take the baptism clothes, white clothes, take out a strip, a single piece, and burn it to protect the child, so the child does not have this disease [seizure]. If the child has the attack, the whole disease, which is in the child’s sweat, remains on the clothes. You take off your clothes before the child returns from the crisis and burns all the clothes for the child to have, takes them away, and burns no longer.			
DCS—Central Idea D: The people here use plants. We have all kinds of plants to use when you need them. And we only take it out when we need it. We also buy them at the city fair. There are also herbalists nearby. Many children are cured with a plant from Sítio Arruda. Here, people use more natural medicine. The plants cure almost everything, and there has a plant for that too [seizure]. We know when the child is going to attack, keep sweating, sweating, getting different. After the attack, you can give tea and some homemade preparations. When I was a little girl, my mom did a lot for me. There is also coconut water, you use it to wash the child’s head [...] but what you do most is tea, and you can use it once or twice, you just can’t put too much, it has to be too little, yet more for children.			

* A participant may present more than one CI in his speech. For this reason, *n* > 19 because the number of times that a given CI was pointed out in the participant’s speech is counted. # Adjusted for the wording of some questions at the time of the interview to allow a better understanding of the community’s residents.

## Data Availability

The data presented in this study are available on request from the corresponding author. The data are not publicly available to protect confidentiality of the research participants.
